# Anti-Neoplastic Activity of Two Flavone Isomers Derived from *Gnaphalium elegans* and *Achyrocline bogotensis*

**DOI:** 10.1371/journal.pone.0039806

**Published:** 2012-06-29

**Authors:** Christan M. Thomas, Robert C. Wood, Jarrett E. Wyatt, Morgan H. Pendleton, Ruben D. Torrenegra, Oscar E. Rodriguez, Sam Harirforoosh, Maria Ballester, Janet Lightner, Koyamangalath Krishnan, Victoria P. Ramsauer

**Affiliations:** 1 Bill Gatton College of Pharmacy, East Tennessee State University, Johnson City, Tennessee, United States of America; 2 Universidad de Ciencias Aplicadas y Ambientales, Bogota, Colombia; 3 Department of Pharmaceutical Sciences, Bill Gatton College of Pharmacy, East Tennessee State University, Johnson City, Tennessee, United States of America; 4 Division of Math Science and Technology, Farquhar College of Arts and Sciences, Nova Southeastern University, Fort Lauderdale, Florida, United States of America; 5 Division of Hematology-Oncology, Department of Internal Medicine, James Quillen College of Medicine, East Tennessee State University, Johnson City, Tennessee, United States of America; Univ of Bradford, United Kingdom

## Abstract

Over 4000 flavonoids have been identified so far and among these, many are known to have antitumor activities. The basis of the relationships between chemical structures, type and position of substituent groups and the effects these compounds exert specifically on cancer cells are not completely elucidated. Here we report the differential cytotoxic effects of two flavone isomers on human cancer cells from breast (MCF7, SK-BR-3), colon (Caco-2, HCT116), pancreas (MIA PaCa, Panc 28), and prostate (PC3, LNCaP) that vary in differentiation status and tumorigenic potential. These flavones are derived from plants of the family *Asteraceae*, genera *Gnaphalium* and *Achyrocline* reputed to have anti-cancer properties. Our studies indicate that 5,7-dihydroxy-3,6,8-trimethoxy-2-phenyl-4H-chromen-4-one (5,7-dihydroxy-3,6,8-trimethoxy flavone) displays potent activity against more differentiated carcinomas of the colon (Caco-2), and pancreas (Panc28), whereas 3,5-dihydroxy-6,7,8-trimethoxy-2-phenyl-4H-chromen-4-one (3,5-dihydroxy-6,7,8-trimethoxy flavone) cytototoxic action is observed on poorly differentiated carcinomas of the colon (HCT116), pancreas (Mia PaCa), and breast (SK-BR3). Both flavones induced cell death (>50%) as proven by MTT cell viability assay in these cancer cell lines, all of which are regarded as highly tumorigenic. At the concentrations studied (5–80 µM), neither flavone demonstrated activity against the less tumorigenic cell lines, breast cancer MCF-7 cells, androgen-responsive LNCaP human prostate cancer line, and androgen-unresponsive PC3 prostate cancer cells. 5,7-dihydroxy-3,6,8-trimethoxy-2-phenyl-4H-chromen-4-one (5,7-dihydroxy-3,6,8-trimethoxy flavone) displays activity against more differentiated carcinomas of the colon and pancreas, but minimal cytotoxicity on poorly differentiated carcinomas of these organs. On the contrary, 3,5-dihydroxy-6,7,8-trimethoxy-2-phenyl-4H-chromen-4-one (3,5-dihydroxy-6,7,8-trimethoxy flavone) is highly cytotoxic to poorly differentiated carcinomas of the colon, pancreas, and breast with minimal activity against more differentiated carcinomas of the same organs. These differential effects suggest activation of distinct apoptotic pathways. In conclusion, the specific chemical properties of these two flavone isomers dictate mechanistic properties which may be relevant when evaluating biological responses to flavones.

## Introduction

There is a group of medicinal plants commonly known in the Andean regions of South America as vira-viras. These plants belong to the family *Asteraceae*, tribe *Inuleae,* and to the genera *Gnaphalium, Achyrocline,* and *Gamochaeta* [1], [2], [3]. They are annuals or perennials that grow between 2000 and 3200 meters above sea level, to an average height of 1.5 meters. Because of their morphological characteristics they can easily be confused with species belonging to different genera. The medicinal use of these plants is not limited to South America, since similar species belonging to these genera grow in various parts of the world, and are commonly used for diverse medicinal purposes. Thus, some *Gnaphalium* species are used in poultices to tend wounds, as a hemostatic, to fight infections, or ease inflammation. For the cure of cancer, it is recommended in the Andean regions of South America, the hot beverage obtained by decoction of *Gnaphalium purpureum* L., and *Gnaphalium elegans H.B.K.* [4]. Ethnobotanical data provide valuable information to further the research and identification of compounds derived from plants that have been traditionally used in medicinal preparations by various cultures. The search for compounds responsible for the therapeutic effects reported by ethnobotanical studies begins with the sequential fractionation of extracts derived from specific parts of the plant, followed by the isolation of molecules present in fractions which display biological activity. Among the molecules with highest therapeutic potential, flavonoids stand out for their extensive range of pharmacological and biological activities. In vitro and in vivo studies have shown that flavonoids possess not only cardioprotective [5], [6] anti-inflammatory [7], [8], antimicrobial [9], [10], antioxidant, anti mutagenic, and anti-tumorigenic activities [11], [12], but are among the most widespread secondary plant metabolites. Flavonoids potential as antitumor agents is based on mechanisms that include induction of apoptosis, cell cycle arrest, and modulation of protein kinase activities [13], [14], [15] on cancer cells. Several of these compounds and their derivatives have been studied in clinical trials, as is the case of genistein, flavopiridol, catechin, and quercetin [16].

**Figure 1:**
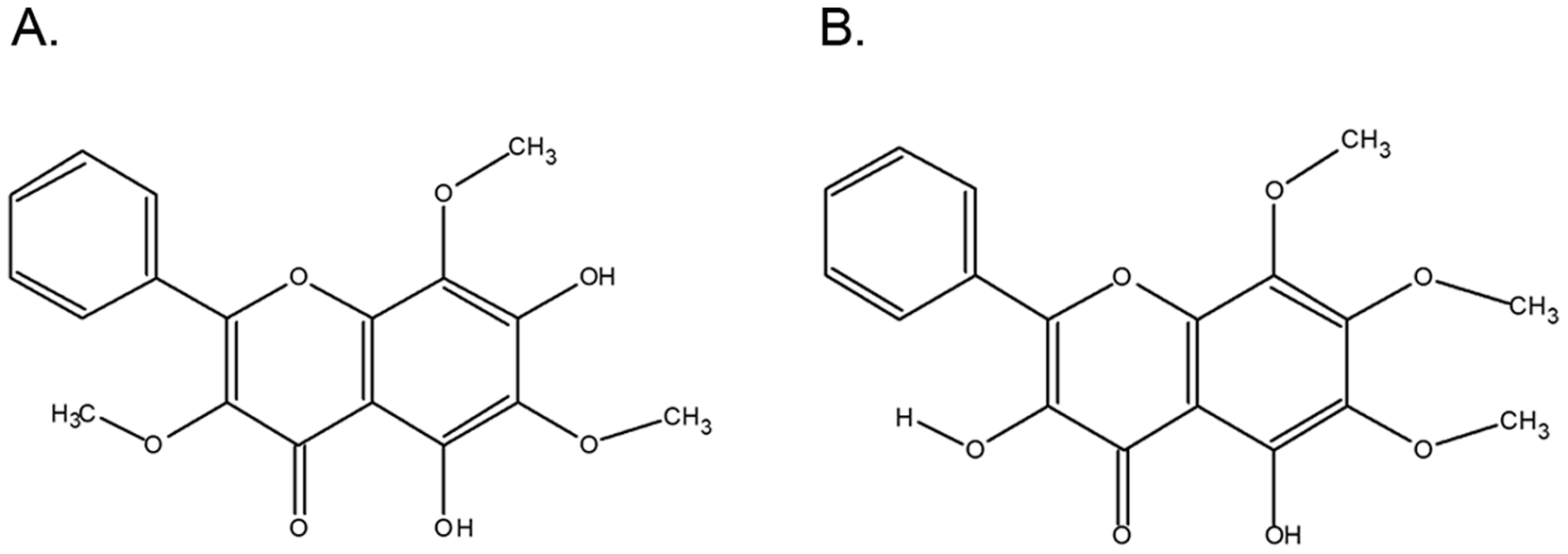
Molecular structures of flavones A and B. A. Flavone A was identified by its physical and spectroscopic properties as 5,7 dihydroxy-3,6,8 trimethoxyflavone. B. Flavone B was identified by its physical and spectroscopic properties as 3,5-dihydroxy-6,7,8-trimethoxyflavone.

In this study we present the antineoplastic effects of two known flavone isomers: 5,7-dihydroxy-3,6,8-trimethoxy-2-phenyl-4H-chromen-4-one (5,7-dihydroxy-3,6,8-trimethoxy flavone or flavone A), and 3,5-dihydroxy-6,7,8-trimethoxy-2-phenyl-4H-chromen-4-one (3,5-dihydroxy-6,7,8-trimethoxy flavone or flavone B) isolated from *Gnaphalium elegans* [3] and *Achyrocline bogotensis* [1] respectively. The anticancer activities of these compounds were tested on cells derived from cancers of the colon, pancreas, breast, and prostate with varying tumorigenic and diferentiation status. The cells were treated using concentrations between 5–80 µM, which is considered to be the range where most flavonoids exert physiological activity [13]. The flavones were isolated from bioactive fractions obtained from the respective plants, then characterized and tested for purity. Because these compounds cytotoxic activity is known to specifically target cancer cells while sparing normal, healthy cells [17], [18], we hypothesized that these flavone isomers may have differential antineoplastic activities based on their structure and the tumorigenic potential of the targeted cells, while minimally affecting normal cells.

**Figure 2:**
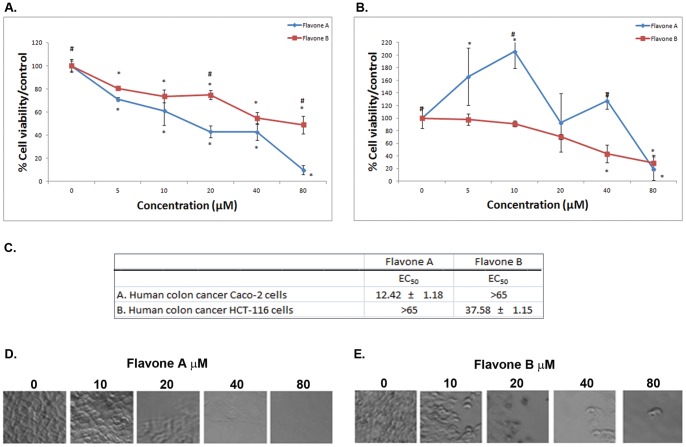
Comparison of the effects of flavone A and flavone B on human colon cancer Caco-2 and HCT-116 cells. The effects of flavone A and flavone B on the more differentiated colon cancer Caco-2 cells (A), and the poorly differentiated colon cancer HCT-116 cells (B) were determined by MTT assay and are represented as a percent of the control absorbance at a wavelength of 570 nm. All data were collected at 24 h after treatment. Data shown are from representative experiments (n = 3). Values are expressed as mean ± SE, * p<0.05, significant difference between control and other concentrations for each flavone. # p<0.05, significant difference between flavone A and flavone B treated cells at similar concentrations. C. Half maximal effective concentration (EC_50_) ± SE for flavone A and flavone B treatment on colon carcinoma and cells. The values were estimated by non-linear regression analysis. D. Representative phase contrast images of colon carcinoma Caco-2 cells and colon carcinoma HCT-116 (E.) cells, 24 hours after treatment with flavone A and flavone B at 5, 10, 20, 40 and 80 µM, or treated with vehicle (0 µM of flavone A or B).

## Materials and Methods

### Cell Culture

Well-differentiated and poorly-differentiated cell lines that originated in multiple tissue sites were obtained from the American Tissue Type Culture Collection (ATCC; Manassas, VA) including: colon (Caco-2, HCT-116), pancreatic (MiaPaca), breast (MCF-7, SK-BR3), and prostate (LNCaP, PC-3) cell lines. Human colon normal CCD-112 coN fibroblasts were also obtained from the ATCC. Tumor cell lines, and normal fibroblasts were grown in tissue culture according to ATCC instructions.

**Figure 3:**
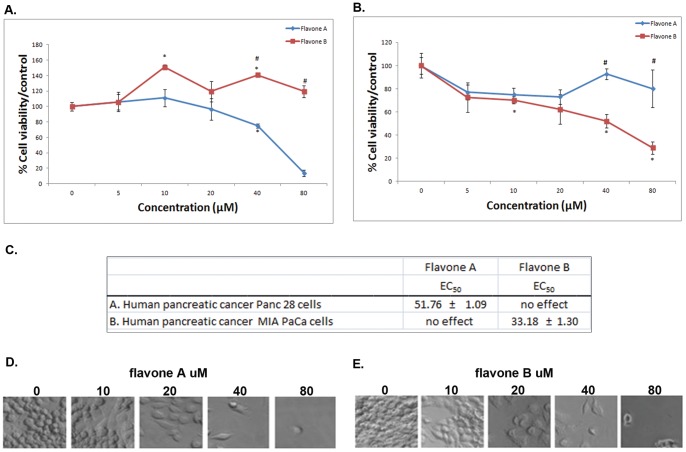
Comparison of the effects of flavone A and flavone B on pancreatic cancer Panc28 and MIA PaCa cells. The effects of flavone A and flavone B on the more differentiated pancreatic cancer Panc28 (A), and the poorly differentiated pancreatic cancer MIA PaCa cells (B) were determined by MTT assay and are represented as a percent of the control absorbance at a wavelength of 570 nm. All data were collected at 24 h after treatment. Data shown are from representative experiments (mean ± SE, n = 3). * p<0.05, significant difference between control and other concentrations for each flavone. # p<0.05, significant difference between flavone A and flavone B treated cells at similar concentrations. C. Half maximal effective concentration (EC_50_) ± SE for flavone A and flavone B treatment on Panc28 and MIA PaCa cells. The values were estimated by non-linear regression analysis. D. Representative phase contrast images of pancreatic cancer Panc28 and MIA PaCa cells (E.) 24 hours after treatment with flavone A and flavone B at 5, 10, 20, 40 and 80 µM, or treated with vehicle (0 µM of flavone A or B).

The Panc-28 cell line was a gift from Dr. Paul Chiao (University of Texas M. D. Anderson Cancer Center, Houston, TX) [19], and was grown in tissue culture in the same manner as pancreatic cell line Mia PaCa-2, in DMEM with hi-glucose media (GIBCO/Invitrogen, Carlsbad, CA) supplemented with 10% serum and penicillin/streptomycin. All cells were seeded and allowed to reach 75% confluency before treatment with flavone A, B or vehicle (DMSO at a final maximum concentration of 0.01%).

**Figure 4:**
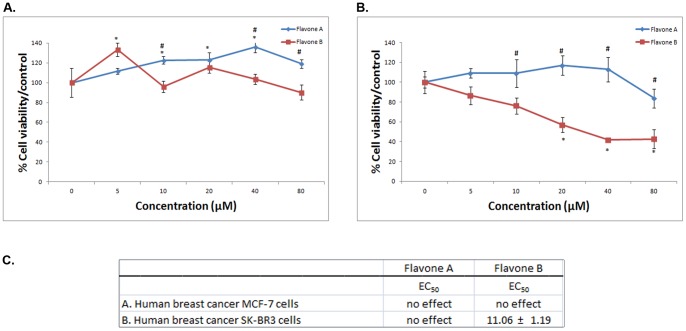
Comparison of the effects of flavone A and flavone B on breast cancer MCF7 and SK-BR3 cells. The effects of flavone A and flavone B on the more differentiated breast cancer MCF7 (A), and the poorly differentiated breast cancer Sk-BR3 cells (B) were determined by MTT assay and are represented as a percent of the control absorbance at a wavelength of 570 nm. All data were collected at 24 h after treatment. Data shown are from representative experiments (mean ± SE, n = 3). * p<0.05, significant difference between control and other concentrations for each flavone. # p<0.05, significant difference between flavone A and flavone B treated cells at similar concentrations. C. Half maximal effective concentration (EC_50_) ± SE for flavone A and flavone B treatment on MCF7 and SK-BR3 cells. The values were estimated by non-linear regression analysis.

### Procedure to obtain 5, 7-dihydroxy-3, 6, 8-trimethoxy flavone (flavone A)

1.5 kg of *G. elegans* dried flowers were extracted with CHCl_3_. The extract was concentrated by dry vacuum, disolved in methanol and filtered to eliminate fats and hydrocarbons. The extract was concentrated and disolved in C_6_H_6_, followed by silica gel chromatography using C_6_H_6_∶Me_2_CO (19∶1) as eluent. 50 mg of the flavonoid was purified from fractions 12 through 18 by crystallizations in hexane. The compound was identified by its physical and spectroscopic properties as 5,7 dihydroxy-3,6,8 trimethoxy flavone, mp 170–171°C [3], 1H NMR (300 MHz) 3.86 (3H,s), 3.97 (3H,s), 4.20(3H,s), 7.50–7.6 (3H,m), 8.08–8.16 (2H,m) [3].

**Figure 5:**
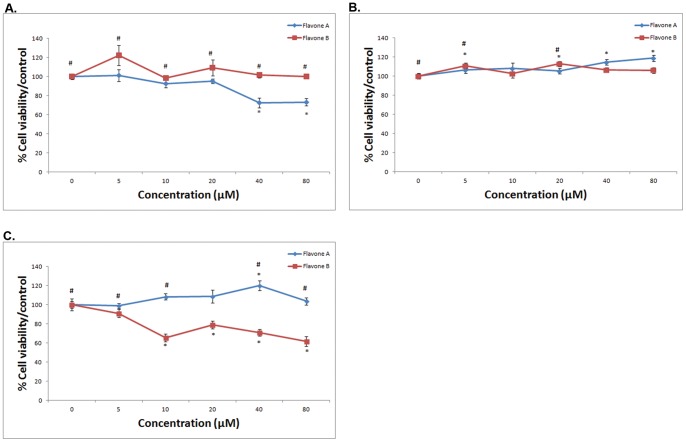
Absence of significant effect on cell viability of flavone A and flavone on prostate cancer LNCaP and PC3 cells as well as on normal human colon fibroblasts (CCD-112 coN). The effects of flavone A and flavone B on the more differentiated prostate cancer LNCaP (A), and the less differentiated PC3 cancer cells (B) were determined by MTT assay and are represented as a percent of the control absorbance at a wavelength of 570 nm. All data were collected at 24 h after treatment. Data shown are from representative experiments (mean ± SE, n = 3). * p<0.05, significant difference between control and other concentrations for each flavone. # p<0.05, significant difference between flavone A and flavone B treated cells at similar concentrations. In the same manner as described above, the effects of flavone A and flavone B normal human colon fibroblasts (C) were determined by MTT assay.

### Procedure to obtain 3, 5-dihydroxy-6, 7, 8-trimethoxy flavone (flavone B)

200 g of *A. bogotensis* fresh leaves were submerged in CHCl_3_ for 20 minutes. The extract was filtered, concentrated and disolved in hot methanol. The cold extract was filtered to eliminate fats and was concentrated once again; the obtained solid was disolved in hot hexane. 100 mg of the purified flavonol was obtained by successive recrystallizations in hexane. The compound was identified by its physical and spectroscopic properties as 3,5-dihydroxy-6,7,8-trimethoxy flavone, mp149–150°C [1], 1H NMR (300 MHz) 3.86 (3H,s), 3.97 (3H,s), 3.99 (3H,s), 4.12 (3H, s), 7.30–7.45 (3H,m), 8.70–8.82 (3H,m), 11.46 (1H,s) [1].

**Figure 6:**
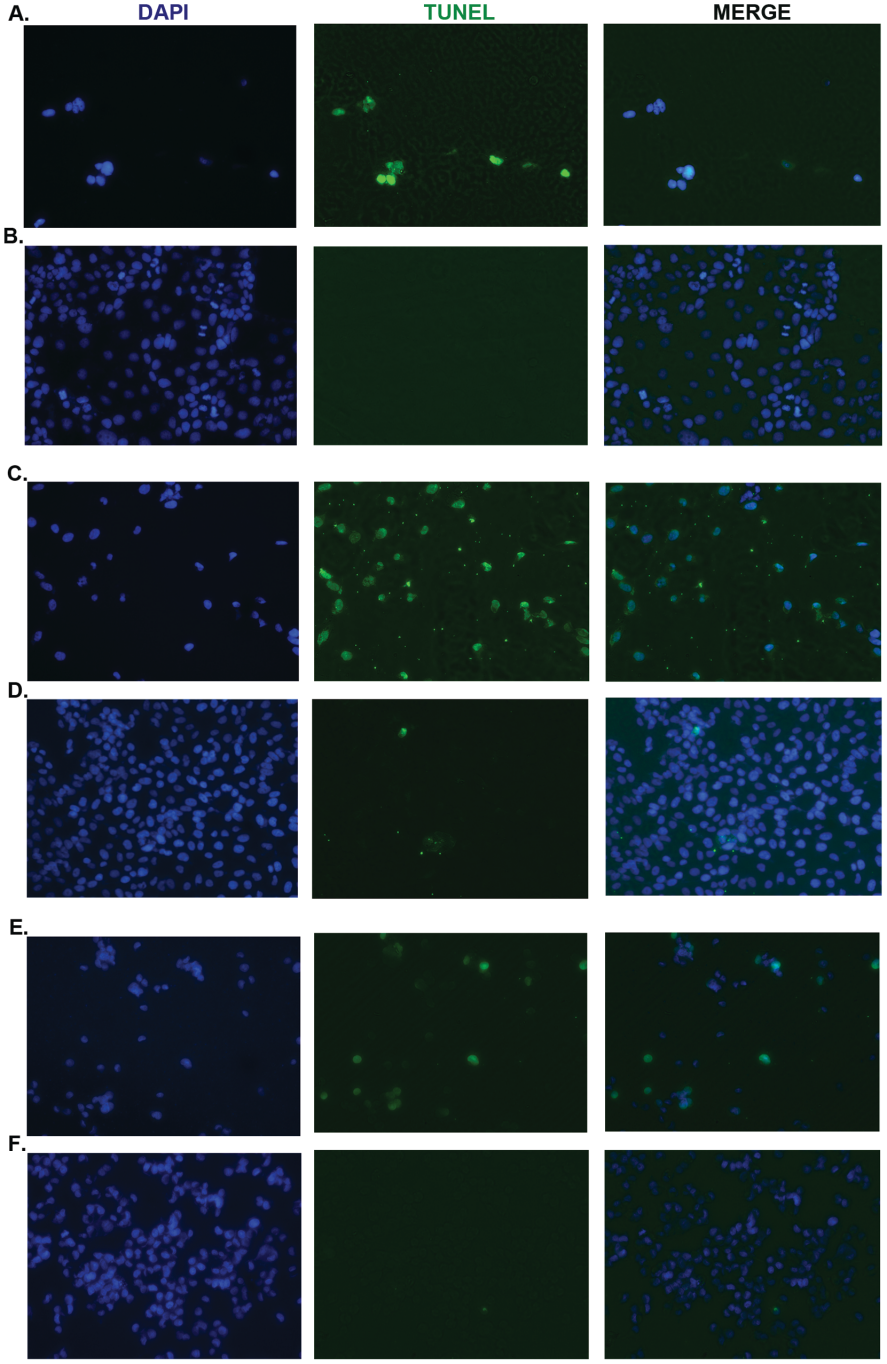
TUNEL assay. A. Apoptotic effect of flavone A at a concentration of 40 µM, on the more differentiated colon cancer Caco-2 cells, as determined by TUNEL assay (green channel) 90 minutes after treatment. DAPI (blue channel) is used to locate the nuclei of the cells. B. Colon Caco-2 cells treated with vehicle only (DMSO at a final concentration of 0.01%) served as a control. TUNEL assay was conducted 90 minutes after treatment. C. Activation of apoptosis on the more differentiated pancreatic cancer Panc28 cells by flavone A at a concentration of 40 µM, as determined by TUNEL assay (green channel) 90 minutes after treatment. DAPI is used to locate the nuclei of the cells. D. As a control, pancreatic cancer Panc28 cells were treated with vehicle only (DMSO at a final concentration of 0.01%) and TUNEL assay was carried out 90 minutes after treatment. E. Apoptotic effect of flavone B at a concentration of 40 µM, on poorly-differentiated pancreatic cancer MIA PaCa cells, as determined by TUNEL assay (green channel) 90 minutes after treatment. DAPI is used to locate the nuclei of the cells. F. Pancreatic cancer MIA PaCa cells were treated with vehicle only as a control (DMSO at a final concentration of 0.01%), and TUNEL assay was conducted 90 minutes after treatment.

#### MTT assay

Cells were seeded at a density of 4000/well in 48 well plates, grown overnight and treated with either vehicle, flavone A or flavone B in concentrations of 5, 10, 20, 40, 60, 80 µM; dissolution vehicle was dimethyl sulfoxide to yield a maximum final concentration of 0.01% in the treated well (Sigma-Aldrich, St. Louis, MO). After 24 hours of incubation 3-(4, 5-methyl-thiazol-2-yl)-2, 5-diphenyl-tetrazolium bromide (MTT) was added at 100 µg/well for 3 hours (Invitrogen). Formazan products were solubilized with acidified 2-propanol and optical density was measured at 570 nm using a Cary 50 (Varian, Palo Alto, CA). All experiments were done in triplicate. Data from assays displaying decrease of cell viability ≥50%, were evaluated by nonlinear regression analysis (GraphPad Prism, La Jolla, CA), and represented as the effective concentration required to decrease 50% of cell viability (EC_50_). Phase contrast images of the treated cells were obtained using a Zeiss Axio Observer inverted microscope, equipped with a Zeiss AxioCam CCD camera.

#### TUNEL assay

Cells at 75% confluency were treated with 40μM flavone A, flavone B, or vehicle (DMSO at a maximum final concentration of 0.01%) for 90 minutes. The cells were fixed with 4% paraformaldehyde and permeabilized with 0.1% sodium citrate and 0.1% Triton X. DNA fragmentation was determined by TdT-mediated dUTP nick end labeling (TUNEL) as described by the manufacturer (Roche Applied Science, Mannheim, Germany). Fluorescent images were obtained using an EVOS fluorescent microscope (AMG, Bothell, WA).

### Data Treatment and Statistical Analysis

Data were analyzed for significant difference using two-way ANOVAs with compound and concentration as factors. Significant main effects were followed with Bonferroni-Holm post hoc tests (SAS 9.2; SAS Institute, Cary, NC). Statistical significance was set at p<0.05.

## Results

### Flavone A and flavone B are known isomers

Flavone A derived from *Gnaphalium elegans* and previously described by Torrenegra et al., [3] as 5,7 dihydroxy-3,6,8 trimethoxy flavone, and flavone B derived from *Achyrocline bogotensis* and previously described by Torrenegra et al., [1] as 3,5-dihydroxy-6,7,8-trimethoxy flavone, are isomers as shown in figure 1. The hydroxyl group in position 7, and the methoxyl group in position 3 in flavone A are switched with a methoxy and hydroxyl groups respectively in flavone B.

### Flavone A but not flavone B greatly decreases cell viability of Caco-2 human colon cancer cells

To determine whether the flavones where cytotoxic to colon cancer, the cells (figures 2A and B) were treated with either one of the compounds. Flavone A effectively decreased cell viability of the more differentiated Caco-2 cells in a concentration-dependant manner, as indicated by MTT assays. Flavone B exerts a markedly lesser effect on these cells as shown in figure 2A. This is evidenced by a half maximal effective concentration (EC_50_), for flavone A of 12.42 µM versus 74.82 µM for flavone B (figure 2C). Images of Caco-2 cells treated with increasing concentrations of flavone A, show progressive cell death (figure 2D).

### Flavone B but not flavone A greatly decreases cell viability of poorly differentiated HCT-116 human colon cancer cells

Flavone B effectively decreased cell viability of HCT-116 cells as indicated by MTT assays, while flavone A has a minimal effect, as shown in figure 2B. The half maximal effective concentration (EC50) for flavone B is 37.50 µM, and 69.99 µM for flavone A (figure 2C). Images of colon carcinoma (HCT-116) cells treated with increasing concentrations of flavone B, display a gradual decrease of cell viability (figure 2E).

### Flavone A but not flavone B is cytotoxic to Panc28 human pancreatic cancer cells

To determine whether the flavones where cytotoxic to pancreas cancer cells (figures 3A and B), we treated the cells with either one of the compounds. Flavone A significantly decreased the cell viability of the more differentiated Panc28 cells as indicated by MTT cell survival assays, whereas flavone B had no effect at this concentration range (figure 3A). The half maximal effective concentration (EC_50_) for flavone A on Panc28 cells is 51.76 µM (figure 3C). Images of Panc28 cells treated with flavone A depict progressive cell death at increasing concentrations (figure 3D).

### Flavone B but not flavone A is cytotoxic to poorly differentiated MIA PaCa human pancreatic cancer cells

Based on MTT assays, flavone B is cytotoxic to MIA PaCa cells, but not flavone A (figure 3B). The half maximal effective concentration (EC_50_) for flavone A on MIA PaCa cells is 33.18 µM (figure 3C). Images of decreasing cell viability of pancreatic carcinoma (MIA PaCa) cells treated with increasing concentrations of flavone B for 24 hours are shown in figure 3E.

### Neither flavone A nor flavone B has an effect on the more differentiated MCF-7 human breast cancer cells; but flavone B has a marked inhibitory effect on the poorly differentiated SK-BR3 breast cancer cells

To determine whether the flavones where cytotoxic to breast cancer cells, we treated the cells with either one of the compounds. MTT cell viability assays of estrogen receptor and progesterone receptor positive (ER^+^/PR^+^) MCF7 cells (figure 4A) treated with flavones A and B demonstrate the absence of an effect on cell survival. Figure 4B shows flavone B effectively decreases cell viability on receptor negative (EṜ/PṜ), poorly differentiated SK-BR3 human breast cancer cells, with a half maximal effective concentration of 11.06 µM (figure 4C).

### Neither Flavone A nor flavone B is cytotoxic to human prostate carcinoma LNCaP and PC3 cells

To determine whether the flavones where cytotoxic to prostate cancer cells, we treated the cells with either one of the compounds. Flavone A and flavone B failed to decrease cell viability of the more differentiated [20]–[21] androgen-responsive LNCaP cells (figure 5A), as well as the poorly differentiated [22] PC3 androgen receptor negative cells (figure 5B) as indicated by MTT assay.

### Neither flavone A nor flavone B is cytotoxic to normal fibroblasts

To determine whether the flavones were cytotoxic to normal cells, we treated normal fibroblasts with either one of the compounds. Cell viability of normal colon fibroblasts CCD-112 coN, is unaffected by either flavone as shown by MTT assay after a 24 hour treatment with increasing concentrations of the compounds between 5–80 µM (figure 5C).

The apoptotic effects of flavone A and flavone B, were confirmed via TUNEL assay as shown on figure 6 on Caco-2, Panc 28, and MIA PaCa cells.

## Discussion

*Gnaphalium elegans* and *Achyrocline bogotensis* were selected for their medicinal properties reported in ethnobotanical studies [4]. These plants are indigenous to the Andean region of South America, but have successfully undergone preliminary domestication studies. The flavones presented in this study are not exclusive to plants native to this geographic location. Flavone A has been isolated from *Ainsliaea henryi*, native to the southern and western portions of China [23]. Tomas-Lorente et al., has reported that flavone A was extracted from *Helichrysum decumbens* along with two other methylated flavonoids [24] Flavone B has been found in *Helichrysum graveolens* [25], *Helichrysum odoratissimum* [26] and *Helichrysum compactum* [27]. The plant extracts of the latter demonstrated to have antibacterial and antioxidant activities. Medicinal plants belonging to the genus *Helichrysum*, traditionally have been used in Rwanda and other African countries for their antibacterial, antitussive, and sedative properties. Research on the therapeutic use of flavonoids has advanced greatly in the last ten years, and some compounds are being tested in clinical trials.

Our findings identify flavone A and its isomer flavone B, as potential anti-tumor agents. Indeed, these compounds display cytotoxic activity against cell lines that have been categorized as being highly tumorigenic. High-expression levels of Aldehyde dehydrogenase (ALDH) is regarded as a very specific marker used in the detection of cancer-initiating cells as subpopulations in tumors, [28], [29], [30], [31], [32], [33] as well as in the determination of tumorigenic status in established cancer cell lines. Flavone A and flavone B cytotoxic activity is directed towards highly tumorigenic cells that express high levels of ALDH: Caco-2 and HCT-116 [34], [35], as well as pancreatic cancer Panc28 and MIA PaCa, [36], and breast cancer SK-BR3 [37] cells. On the contrary, Flavone A and B activities were observed to be significantly diminished in less tumorigenic cells, characterized by low expression levels of ALDH: breast cancer MCF7 cells [34], [37], and prostate cancer PC3and LNCaP cells [38].

Cancer cell lines display variability in regards to differentiation status. Thus, more differentiated cells can be distinguished from poorly differentiated cells by the presence of specific polarity and differentiation markers, as well as doubling time. Among these highly tumorigenic cells upon which the flavones activity is targeted, we find that flavone A has a preferential effect on the more differentiated colon cancer Caco-2 [39] cells, and pancreatic cancer Panc28 cells. It is important to note that the latter has been classified as poorly differentiated when polarity markers are compared with better differentiated pancreatic cancer cells such as Capan-1. However, it is the general consensus that Panc28 cells possess a higher differentiation status than MIA PaCa cells, specifically when doubling time is considered [40]. Our results suggest that flavone A has a preference for the more-differentiated colon carcinoma Caco-2 cells and pancreatic cancer Panc28 cells as compared to greatly diminished activity on poorly differentiated cells. On the contrary, flavone B displays preferential activity towards poorly differentiated cancer cells of the colon (HCT-116), pancreas (MIA PaCa) and cadherin negative breast cancer SK-BR3 cells [41].

In line with previous reports [13], neither flavone A nor flavone B is cytotoxic to normal cells. However, it has been shown that this lack of cytotoxic activity on normal cells while targeting malignant cells, has several exceptions as is the case of the hydroxyflavones luteolin and apigenin. Recent studies conducted on methoxylated hydroxyflavones suggest that the presence of sequential methoxylated groups, as observed in flavone B, may dictate lack of cytotoxic activity on normal cells [42]. Extensive studies indicate that certain substitution patterns in flavones may increase their anticancer activity. These observations may benefit from additional information in regards to characteristics that may serve to categorize cancer cell lines prone to anticancer activity. In these flavone isomers, the positions of –OH and –OCH_3_ groups not only alter the conformation but also the charge delocalization of the compounds, which may be relevant to cellular uptake. In flavone A, both 5- and 7-OH can resonate with the carbonyl, but the 5-OH next to the carbonyl has one more resonance structure. In flavone B, the 5-OH can resonate with the carbonyl but not the 3-OH. While optimization of these structures is yet to be obtained and analyzed, charge delocalization and resonance as a result of the position of substituents are likely to influence anti cancer activities.

At the cellular level, our results suggest an appreciable degree of neoplastic specificity. The preferential effect of these two isomers on cancers with varying differentiation status, suggest a bifurcation in the mechanisms of action of these compounds that may afford new insights on the study of these malignancies. Specific and effective growth inhibition of cancer cells by these compounds may provide alternative therapies urgently needed in highly aggressive neoplasias such as pancreatic cancer which continues to have a very low five year survival rate.

Recent reports have shown the use of flavonoids to augment the effectiveness of existing oncology medications [28], [39], [43]. The process by which these compounds are able to effectively induce the activation apoptosis in cancer cells is also of great interest [30], [31]. The differential effects described in this study, suggest activation of distinct apoptotic pathways. Initial studies on the mechanism of action of these compounds, however, indicate that both induce apoptosis via the activation of the intrinsic caspase cascade. TUNEL assay which specifically detects apoptosis was carried out 1.5–4 hours after treatment with the flavones or vehicle, due to parallel occurrence of apoptosis activation and cellular detachment (figure 6). These observations suggest that these compounds may interfere shortly after treatment with signaling pathways associated with cell-matrix and cell-cell adhesion molecules, as apoptosis takes place. Investigation in the mechanisms of activation upstream of the activation of caspases is necessary to better understand the potential use of these compounds in novel antineoplastic therapies.

This study presents two flavone isomers that may be responsible for the anticancer activities associated to *Gnaphalium elegans* and *Achyrocline bogotensis*. We also present evidence that suggest that these compounds display preferential targeting which may depend upon cellular tumorigenic and differentiation status. Further studies are necessary to elucidate the mechanisms responsible for the observed differential effects.

## References

[1] Torrenegra RD, Escarria S, Tenorio E, Achenbach H (1982). Estudio fitoquimico del Achirocline bogotensis.. Rev Latinoam Quim.

[2] Torrenegra R, Pedrozo P, Rojas C, Carrizosa S (1987). Plantas Colombianas del género Gnaphalium (IV) G. rufescens y G. antennarioides.. Revista Latinoamericana de Química.

[3] Torrenegra RD, Escarria S, Raffelsberger B, Achenbach H (1980). 5,7-Dihydroxy-3,6,8-trimethoxyflavone from the flowers of Gnaphalium elegans.. Phytochemistry.

[4] Garcia-Barriga H (1975). Flora Medicinal de Colombia.. Bogota: Imprenta Nacional III.

[5] Perez-Vizcaino F, Duarte J, Jimenez R, Santos-Buelga C, Osuna A (2009). Antihypertensive effects of the flavonoid quercetin.. Pharmacol Rep.

[6] Wang S, Thomas CJ, Dusting GJ, Woodman OL, May CN (2009). 3′,4′-Dihydroxyflavonol improves post-ischaemic coronary endothelial function following 7days reperfusion in sheep.. Eur J Pharmacol.

[7] Garcia-Lafuente A, Guillamon E, Villares A, Rostagno MA, Martinez JA (2009). Flavonoids as anti-inflammatory agents: implications in cancer and cardiovascular disease.. Inflamm Res.

[8] Javed H, Khan MM, Ahmad A, Vaibhav K, Ahmad ME (2012). Rutin prevents cognitive impairments by ameliorating oxidative stress and neuroinflammation in rat model of sporadic dementia of Alzheimer type.. Neuroscience.

[9] Cushnie TP, Lamb AJ (2005). Antimicrobial activity of flavonoids.. Int J Antimicrob Agents.

[10] Fiamegos YC, Kastritis PL, Exarchou V, Han H, Bonvin AM (2011). Antimicrobial and efflux pump inhibitory activity of caffeoylquinic acids from Artemisia absinthium against gram-positive pathogenic bacteria.. PLoS One.

[11] Le Marchand L (2002). Cancer preventive effects of flavonoids–a review.. Biomed Pharmacother.

[12] Dandawate PR, Vyas A, Ahmad A, Banerjee S, Deshpande J (2012). Inclusion Complex of Novel Curcumin Analogue CDF and beta-Cyclodextrin (1∶2) and Its Enhanced In Vivo Anticancer Activity Against Pancreatic Cancer.. Pharm Res.

[13] Lopez-Lazaro M (2002). Flavonoids as anticancer agents: structure-activity relationship study.. Curr Med Chem Anticancer Agents.

[14] Kandaswami C, Lee LT, Lee PP, Hwang JJ, Ke FC (2005). The antitumor activities of flavonoids.. In Vivo.

[15] Kale A, Gawande S, Kotwal S (2008). Cancer phytotherapeutics: role for flavonoids at the cellular level.. Phytother Res.

[16] Wang HK (2000). The therapeutic potential of flavonoids.. Expert Opin Investig Drugs.

[17] Tseng TH, Lee YJ (2006). Evaluation of natural and synthetic compounds from East Asiatic folk medicinal plants on the mediation of cancer.. Anticancer Agents Med Chem.

[18] Liu HL, Jiang WB, Xie MX (2010). Flavonoids: recent advances as anticancer drugs.. Recent Pat Anticancer Drug Discov.

[19] Sclabas GM, Fujioka S, Schmidt C, Evans DB, Chiao PJ (2003). NF-kappaB in pancreatic cancer.. Int J Gastrointest Cancer.

[20] Horoszewicz JS, Leong SS, Chu TM, Wajsman ZL, Friedman M (1980). The LNCaP cell line–a new model for studies on human prostatic carcinoma.. Prog Clin Biol Res.

[21] Lim DJ, Liu XL, Sutkowski DM, Braun EJ, Lee C (1993). Growth of an androgen-sensitive human prostate cancer cell line, LNCaP, in nude mice.. Prostate.

[22] Kaighn ME, Narayan KS, Ohnuki Y, Lechner JF, Jones LW (1979). Establishment and characterization of a human prostatic carcinoma cell line (PC-3).. Invest Urol.

[23] Xiong HP, Wu ZJ, Chen FT, Chen WS (2009). 5,7-Dihydr-oxy-3,6,8-trimethoxy-flavone.. Acta Crystallogr Sect E Struct Rep Online.

[24] Tomás-Lorente F, Iniesta-Sanmartín E, Tomás-Barberán FA, Trowitzsch-Kienast W, Wray V (1989). Antifungal phloroglucinol derivatives and lipophilic flavonoids from Helichrysum decumbens.. Phytochemistry.

[25] Hansel R, Cubukcu B (1972). 3,5-Dihydroxy-6,7,8-trimethoxyflavon aus Helichrysum graveolens.. Phytochemistry.

[26] Van Puyvelde L, De Kimpe N, Costa J, Munyjabo V, Nyirankuliza S (1989). Isolation of flavonoids and a chalcone from Helichrysum odoratissimum and synthesis of helichrysetin.. J Nat Prod.

[27] Suzgec S, Mericli AH, Houghton PJ, Cubukcu B (2005). Flavonoids of Helichrysum compactum and their antioxidant and antibacterial activity.. Fitoterapia.

[28] Lee SH, Ryu JK, Lee KY, Woo SM, Park JK (2008). Enhanced anti-tumor effect of combination therapy with gemcitabine and apigenin in pancreatic cancer.. Cancer Lett.

[29] Shin SY, Hyun J, Yu JR, Lim Y, Lee YH (2011). 5-Methoxyflavanone induces cell cycle arrest at the G2/M phase, apoptosis and autophagy in HCT116 human colon cancer cells.. Toxicol Appl Pharmacol.

[30] Erhart LM, Lankat-Buttgereit B, Schmidt H, Wenzel U, Daniel H (2005). Flavone initiates a hierarchical activation of the caspase-cascade in colon cancer cells.. Apoptosis.

[31] Torres F, Quintana J, Estevez F (2010). 5,7,3′-trihydroxy-3,4′-dimethoxyflavone-induced cell death in human leukemia cells is dependent on caspases and activates the MAPK pathway.. Mol Carcinog.

[32] Middleton E, Kandaswami C, Theoharides TC (2000). The effects of plant flavonoids on mammalian cells: implications for inflammation, heart disease, and cancer.. Pharmacol Rev.

[33] Middleton ET, Morice AH (2000). Breath carbon monoxide as an indication of smoking habit.. Chest.

[34] Neumeister V, Agarwal S, Bordeaux J, Camp RL, Rimm DL (2010). In situ identification of putative cancer stem cells by multiplexing ALDH1, CD44, and cytokeratin identifies breast cancer patients with poor prognosis.. Am J Pathol.

[35] Lin L, Liu Y, Li H, Li PK, Fuchs J (2011). Targeting colon cancer stem cells using a new curcumin analogue, GO-Y030.. Br J Cancer.

[36] Visus C, Wang Y, Lozano-Leon A, Ferris RL, Silver S (2011). Targeting ALDH(bright) human carcinoma-initiating cells with ALDH1A1-specific CD8 T cells.. Clin Cancer Res.

[37] Karna P, Gundala SR, Gupta MV, Shamsi SA, Pace RD (2011). Polyphenol-rich sweet potato greens extract inhibits proliferation and induces apoptosis in prostate cancer cells in vitro and in vivo.. Carcinogenesis.

[38] Schumacher M, Hautzinger A, Rossmann A, Holzhauser S, Popovic D (2011). Potential role of P-gp for flavone-induced diminished apoptosis and increased adenoma size in the small intestine of APC(min/+) mice.. Cancer Invest.

[39] Choi EJ, Kim GH (2009). 5-Fluorouracil combined with apigenin enhances anticancer activity through induction of apoptosis in human breast cancer MDA-MB-453 cells.. Oncol Rep.

[40] Sipos B, Moser S, Kalthoff H, Torok V, Lohr M (2003). A comprehensive characterization of pancreatic ductal carcinoma cell lines: towards the establishment of an in vitro research platform.. Virchows Arch.

[41] Menter DG, Ramsauer VP, Harirforoosh S, Chakraborty K, Yang P (2011). Differential effects of pravastatin and simvastatin on the growth of tumor cells from different organ sites.. PLoS One.

[42] Moghaddam G, Ebrahimi SA, Rahbar-Roshandel N, Foroumadi A (2011). Antiproliferative Activity of Flavonoids: Influence of the Sequential Methoxylation State of the Flavonoid Structure.. Phytother Res.

[43] Singh M, Bhatnagar P, Srivastava AK, Kumar P, Shukla Y (2011). Enhancement of cancer chemosensitization potential of cisplatin by tea polyphenols poly(lactide-co-glycolide) nanoparticles.. J Biomed Nanotechnol.

